# The Role of Glucose Metabolism in *Xenopus* Primitive Myeloid Cell Development and Function

**DOI:** 10.3390/ijms27188175

**Published:** 2026-09-14

**Authors:** Loucas Demetriou, Paris A. Skourides, Neophytos Christodoulou

**Affiliations:** Department of Biological Sciences, University of Cyprus, P.O. Box 20537, 2109 Nicosia, Cyprus; ldemet02@ucy.ac.cy

**Keywords:** primitive myeloid cells, *Xenopus laevis*, glucose metabolism, immunometabolism, hexosamine biosynthetic pathway, glycolysis, pentose phosphate pathway, cell migration, wound healing, embryonic development

## Abstract

Primitive Myeloid Cells (PMCs) arise from the anterior ventral blood islands during *Xenopus laevis* embryogenesis and migrate to colonize embryonic tissues, contributing to wound healing, innate immunity, and development. Because cell movement and growth are metabolically demanding, we investigated whether metabolic pathways regulate PMC behaviour integrating public single-cell RNA-seq data with whole-mount in situ hybridization (WMISH) to profile metabolic gene expression in PMCs, followed by functional assays using pathway-specific inhibitors: 2-deoxy-D-glucose 2-DG (global glucose metabolism), YZ-9 (glycolysis), 6-AN (pentose phosphate pathway, PPP), and ST045849 (hexosamine biosynthetic pathway, HBP). The analyses showed that PMCs express high levels of glycolytic and ancillary metabolic enzymes. Global inhibition impaired PMC colonization and wound recruitment and modestly reduced the size of the specification domain, confirming a requirement for glycolytic flux. PPP and downstream glycolysis inhibition produced only modest, non-significant shifts in recruitment, whereas HBP inhibition abolished wound recruitment and reduced PMC numbers. These findings identify glucose metabolism as a regulator of PMC colonization and immune function, revealing how metabolic reprogramming governs early immune cell behaviour independent of a functional vasculature.

## 1. Introduction

Cell migration is a fundamental process in multicellular organisms, underlying embryonic body-plan formation, tissue homeostasis and the progression of numerous diseases [1,2]. During gastrulation, coordinated movements of cell collectives establish the three germ layers, and specialized populations such as neural crest cells subsequently migrate over long distances to give rise to diverse tissues and organs [3]. In adults, migration remains essential for immune surveillance, epithelial renewal and wound repair [4]. These processes rely on a conserved molecular toolkit—chemokine gradients to guide cells, integrin-based adhesion to extracellular matrix (ECM) components for traction, and cytoskeletal remodelling to generate protrusive forces—whose dysregulation contributes to congenital disorders and, notably, to cancer metastasis [5].

Migration is metabolically costly: nearly half of a motile cell’s adenosine triphosphate (ATP)is consumed by remodelling of the actin cytoskeleton [6], and mitochondria are actively trafficked to the leading and trailing edges of migrating cells to locally fuel actomyosin-based contraction [7,8]. Oxidative phosphorylation (OXPHOS) supports the sustained energy demands of directed migration through dense extracellular matrices [9], yet glycolysis, whose enzymes associate directly with the cytoskeleton, can be spatially coupled to sites of active protrusion and is often indispensable for motility even when OXPHOS is intact [10]. Accordingly, many migratory and invasive cell types favour aerobic glycolysis (the Warburg effect) and increase glucose/pyruvate uptake even in the presence of oxygen [11], and cells preferentially adopt less energetically demanding migratory paths when available [12].

**Figure 1:**
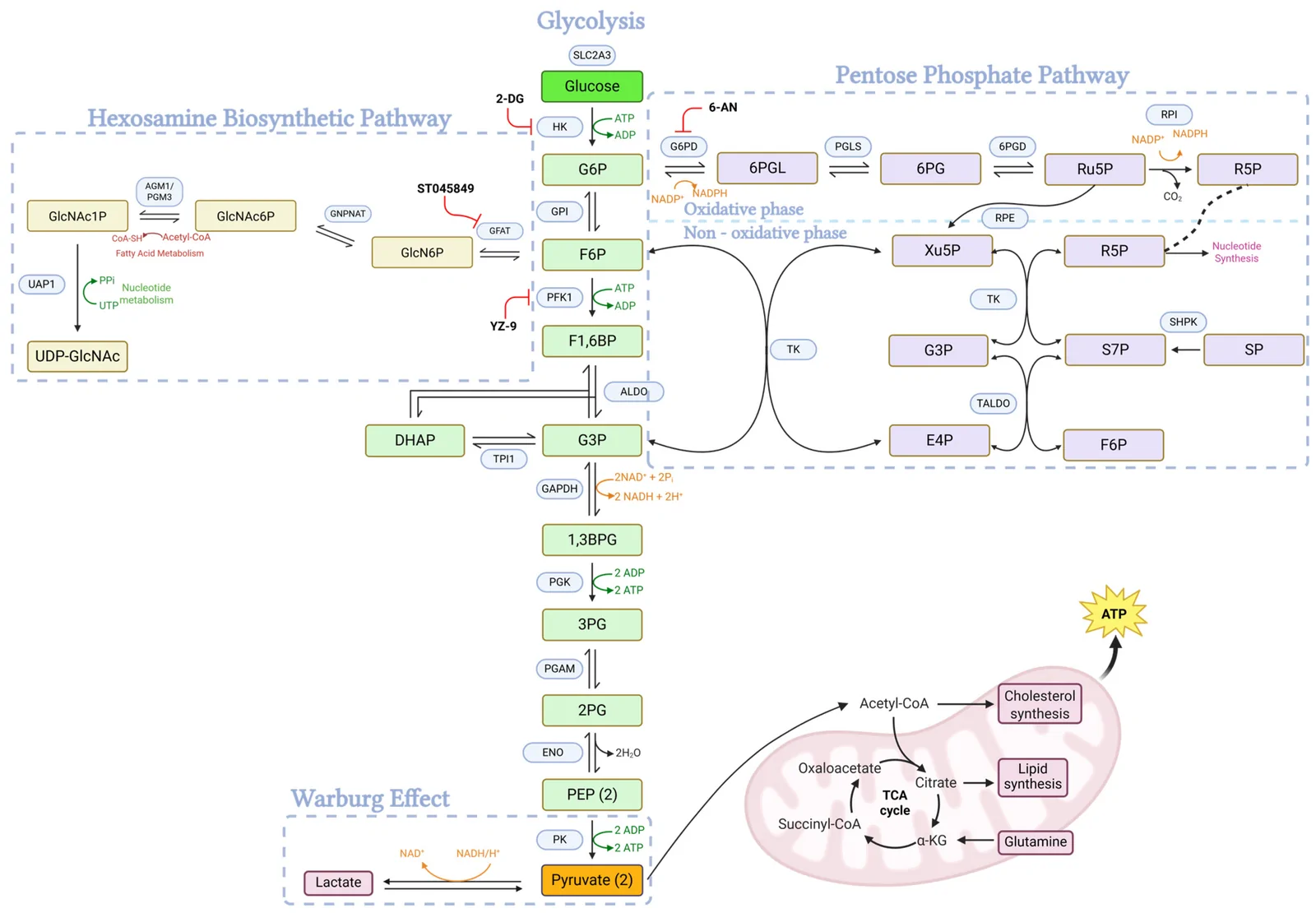
Overview of central glucose metabolism highlighting glycolysis, the pentose phosphate pathway (PPP) and the hexosamine biosynthetic pathway (HBP), with the pharmacological inhibitors used in this study (2-DG, YZ-9, 6-AN and ST045849) and their targets indicated. Created in BioRender. Demetriou, L. (2026) https://biorender.com/tr3qmw8.

Glycolytic intermediates feed two ancillary branches, the pentose phosphate pathway (PPP) and the hexosamine biosynthetic pathway (HBP), which respectively generate NADPH for redox balance and UDP-GlcNAc for protein glycosylation—processes increasingly recognized as important for directional migration and cell–matrix interactions [13,14,15,16] (Figure 1).

This metabolic rewiring is especially prominent in immune cells, where activation triggers a shift toward aerobic glycolysis to meet the energetic and biosynthetic demands of effector function, while resolution-phase and other quiescent populations depend more heavily on OXPHOS [17,18]. Alongside glycolysis, the PPP and HBP are concomitantly upregulated in activated myeloid cells, generating NADPH for the oxidative burst and glycosylation substrates for signalling and adhesion molecules [19]. In classically activated (M1) macrophages, nitric-oxide production disables mitochondrial respiration and repurposes the tricarboxylic acid cycle to generate signalling metabolites such as succinate, which stabilizes HIF-1α to drive IL-1β production [20,21].

Cells of the myeloid lineage constitute the largest population of innate immune effectors, providing continuous immunological surveillance and rapid responses to infection and injury [22,23]. Before the establishment of definitive haematopoiesis in the fetal liver and bone marrow, PMCs arise from extraembryonic or early embryonic blood islands and differentiate into the first population of embryonic macrophages [24]. Despite lacking many effector functions of adult myeloid cells, these primitive macrophages already act instructively during development, clearing apoptotic cells during limb remodelling and cardiac maturation, and guiding tissue patterning in the developing embryo [25,26,27].

*Xenopus laevis* is a well-established model for dissecting early haematopoietic development, owing to its external, synchronous development and large, experimentally tractable embryos [28]. In Xenopus, PMCs arise within the anterior ventral blood islands and are the first blood lineage to differentiate into functional effectors [29,30]; they colonize the embryo and are recruited to wounds with striking efficiency, well before a functional vasculature is established [31]. Despite the well-documented contribution of glucose metabolism to migration and immune effector function in other systems, whether and how specific glucose-metabolic pathways such as glycolysis, the PPP and the HBP govern PMC specification, colonization and injury-induced recruitment in this pre-vascular context remains unknown.

We hypothesized that, as bona fide embryonic immune cells, PMCs undergo metabolic reprogramming that enables their colonization of the embryo and their recruitment to sites of injury. Combining single-cell transcriptomic analysis, whole-mount in situ hybridization and pharmacological inhibition of glycolysis, the PPP and the HBP, we set out to determine which glucose-metabolic pathways are required for PMC development and function. We show that global inhibition of glucose metabolism impairs both PMC colonization and wound recruitment and modestly reduces the size of the PMC specification domain without disrupting the *spib* expression pattern, and that this requirement is only partly explained by classical glycolysis—pointing instead to the hexosamine biosynthetic pathway as a critical, previously unrecognized regulator of PMC recruitment and survival.

## 2. Results

Given the established roles of glycolysis, the pentose phosphate pathway (PPP) and the hexosamine biosynthetic pathway (HBP) in the migration and effector function of other immune cell types [10,17,19], we investigated whether Xenopus primitive myeloid cells (PMCs) deploy these same pathways during their specification, colonization of the embryo, and recruitment to wounds.

### 2.1. Migrating Myeloid Progenitors Express Glycolytic, PPP, and HBP Genes

**Figure 2:**
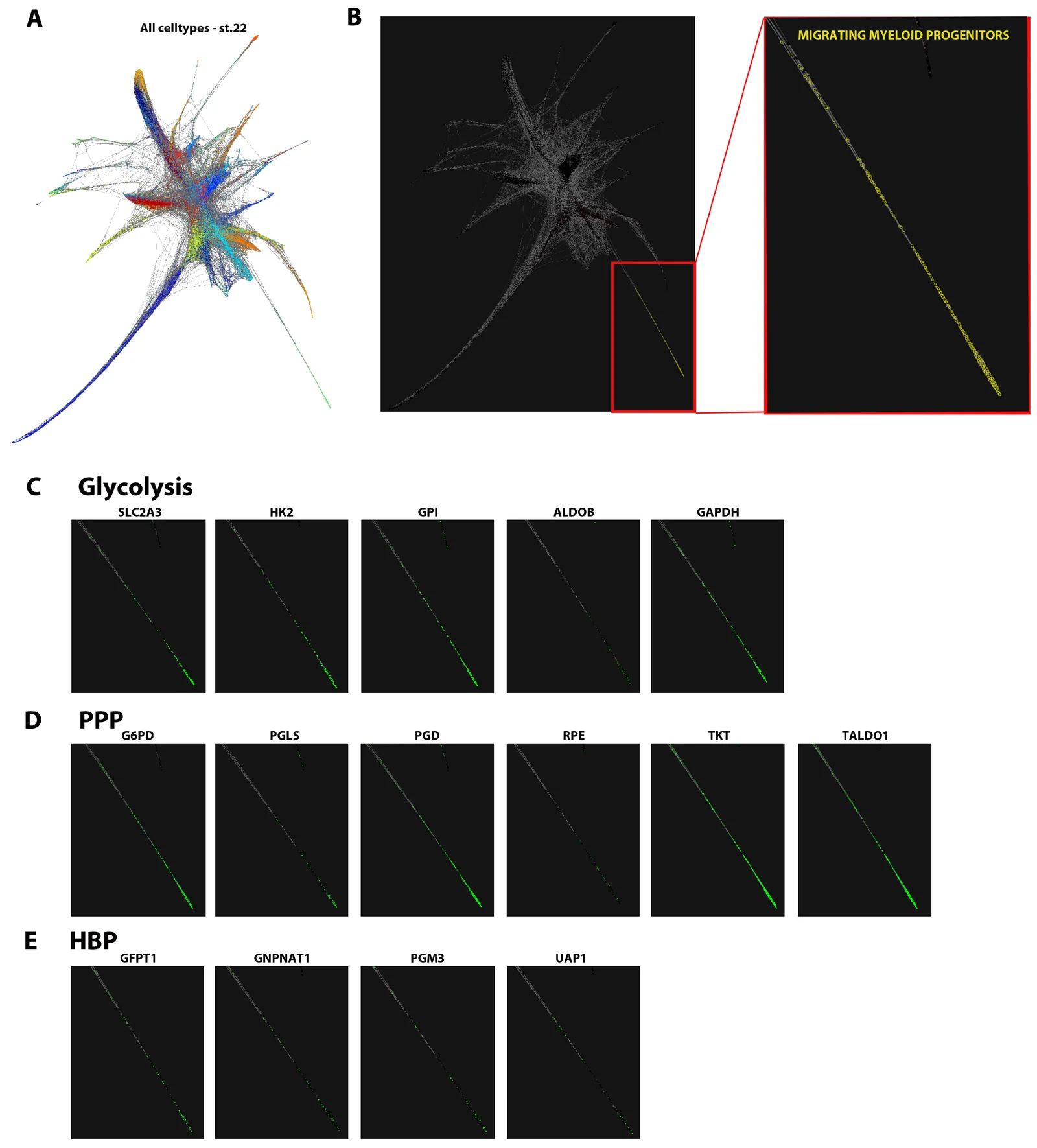
Transcriptomic profiling of migrating myeloid progenitors using the Xenopus Jamboree database. (**A**) All cell types annotated at stage 22. (**B**) Migrating myeloid progenitor cluster isolated for targeted analysis. (**C**–**E**) Expression of glycolytic (**C**), pentose phosphate pathway (**D**) and hexosamine biosynthetic pathway (**E**) genes within this cluster. Each panel shows the single-cell expression of one gene overlaid on the same force-directed embedding; values are already normalized per cell within the source dataset.

Given that genes involved in glycolysis are not uniformly expressed at early developmental stages, we mined a single-cell RNA-sequencing dataset of *Xenopus tropicalis* embryos [32] (Figure 2A) and isolated the annotated migrating myeloid progenitor cluster (Figure 2B), to determine whether PMCs express a distinct glucose-metabolic gene signature. Genes encoding glycolytic enzymes (SLC2A3, HK2, GPI, ALDOB, GAPDH), oxidative and non-oxidative PPP enzymes (G6PD, PGLS, PGD, RPE, TKT, TALDO1) and HBP enzymes (GFPT1, GNPNAT1, PGM3, UAP1) were preferentially detected within this cluster at stages coinciding with the onset of PMC colonization of the embryo (stages 19–20) [31] (Figure 2C–E).

**Figure 3:**
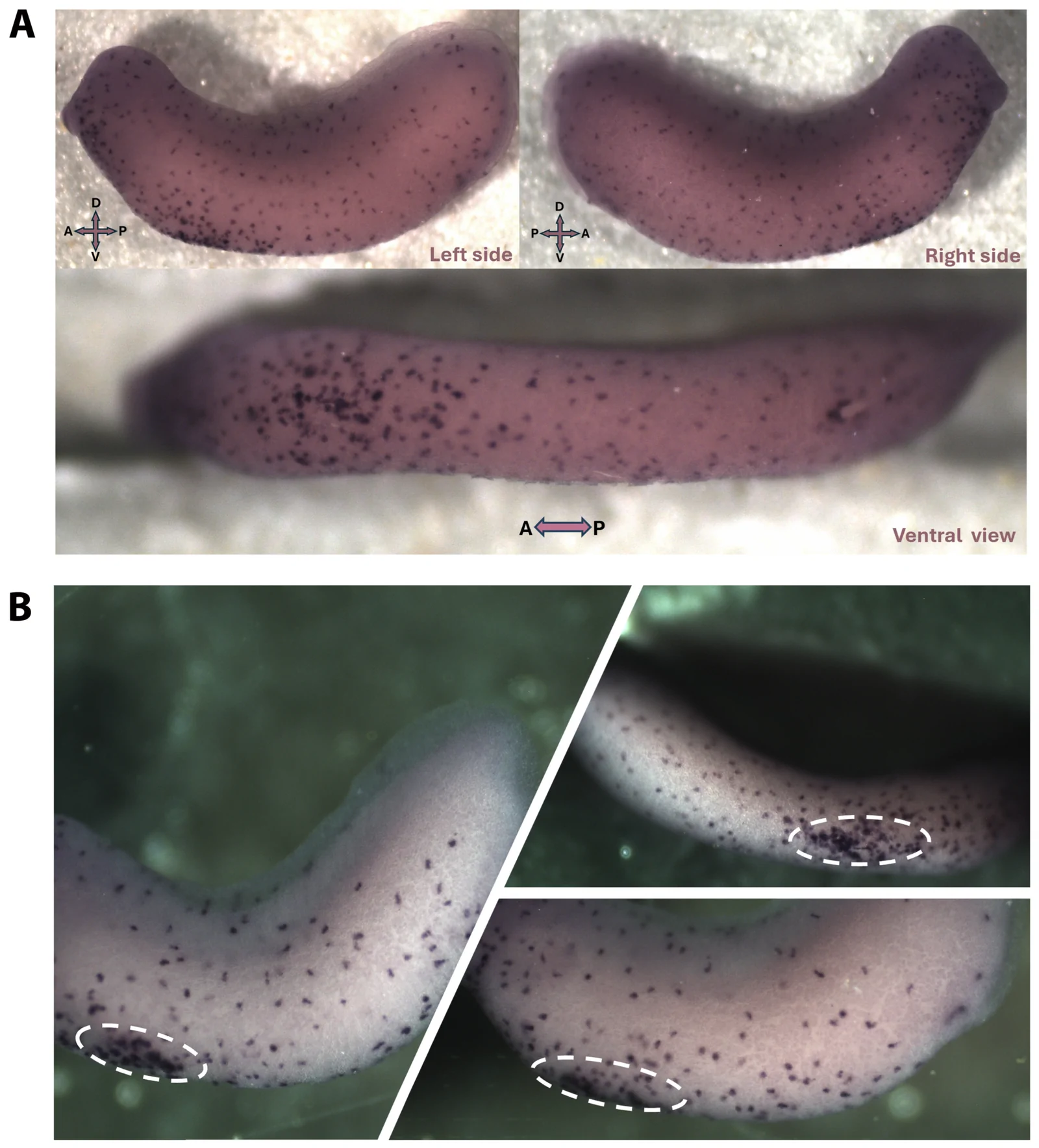
*Spib* expression marks migrating PMCs in Xenopus embryos. WMISH for *spib* showing left and right lateral views (**A**) and a ventral view (**B**) with bilateral domains of expression. Insets (white dashed outline) highlight the anterior ventral blood islands (aVBIs), the site of PMC origin, and individual PMCs that have migrated away from the aVBIs are visible as dispersed cells across the embryo.

To validate this transcriptomic signature in situ, we performed whole-mount in situ hybridization (WMISH) using *spib*, an established PMC-specific transcription factor [31], which labelled PMCs in their characteristic bilateral clusters within the anterior ventral blood islands (aVBIs) and as single dispersed cells along their colonization route (Figure 3). This staining pattern served as a reference template to assess the specificity of subsequent probes.

**Figure 4:**
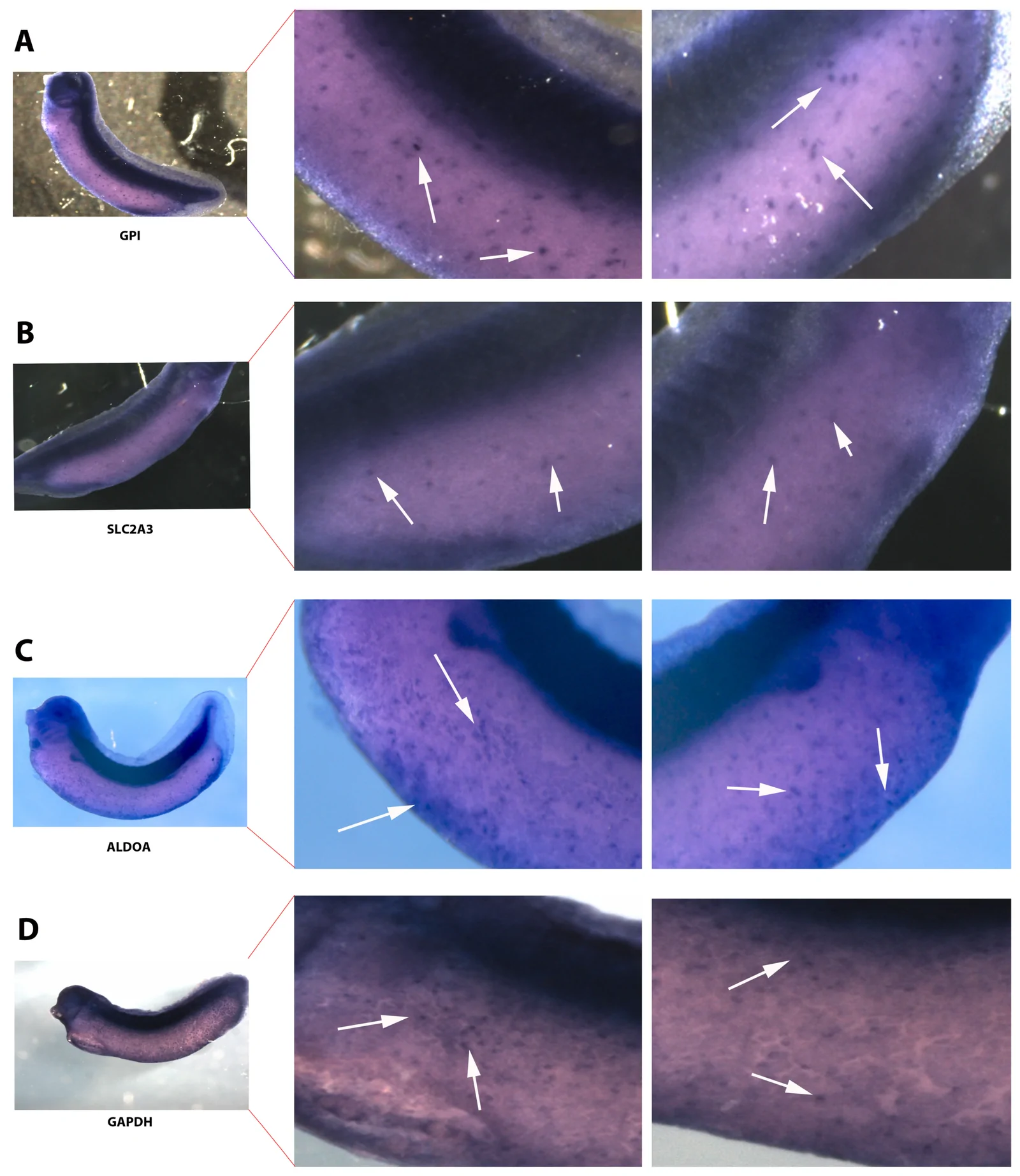
Glycolytic enzymes are expressed in primitive myeloid cells. WMISH for (**A**) GPI, (**B**) SLC2A3, (**C**) ALDOA and (**D**) GAPDH in stage 32 embryos. White arrows indicate PMCs; all four genes show expression patterns overlapping with the *spib* pattern shown in Figure 3.

Genes were selected based on the availability of previously characterized probes with demonstrated specificity, as well as their functional relevance to glycolysis, which we are investigating as a potential contributor to metabolic regulation during progenitor cell migration. WMISH for four glycolytic genes, GPI, SLC2A3, ALDOA and GAPDH, confirmed expression in cells with a scattered, ventral distribution consistent with the *spib* pattern, alongside a strong signal in the somites, where expression is particularly prominent (Figure 4). These data confirm glycolytic gene expression in PMCs from single-cell RNA-seq data. Together, these results indicate that PMCs upregulate glycolysis, PPP and HBP gene programmes during the developmental window in which they colonize the embryo.

### 2.2. Glucose Metabolism Is Dispensable for PMC Specification but Required for Colonization

Given the expression of glycolytic genes in PMCs, we wanted to address the role of glucose metabolism in these cells. Since specific and effective inhibitors are available [33,34,35], coupled with the need for temporal control over activity, we decided to utilize inhibitors for loss-of-function experiments. To ensure that the observed effects of the inhibitors used in this study are specific to cell migration/recruitment and not attributable to broader disruption in cellular identity, we first had to confirm that treatment does not affect the specification of PMCs.

**Figure 5:**
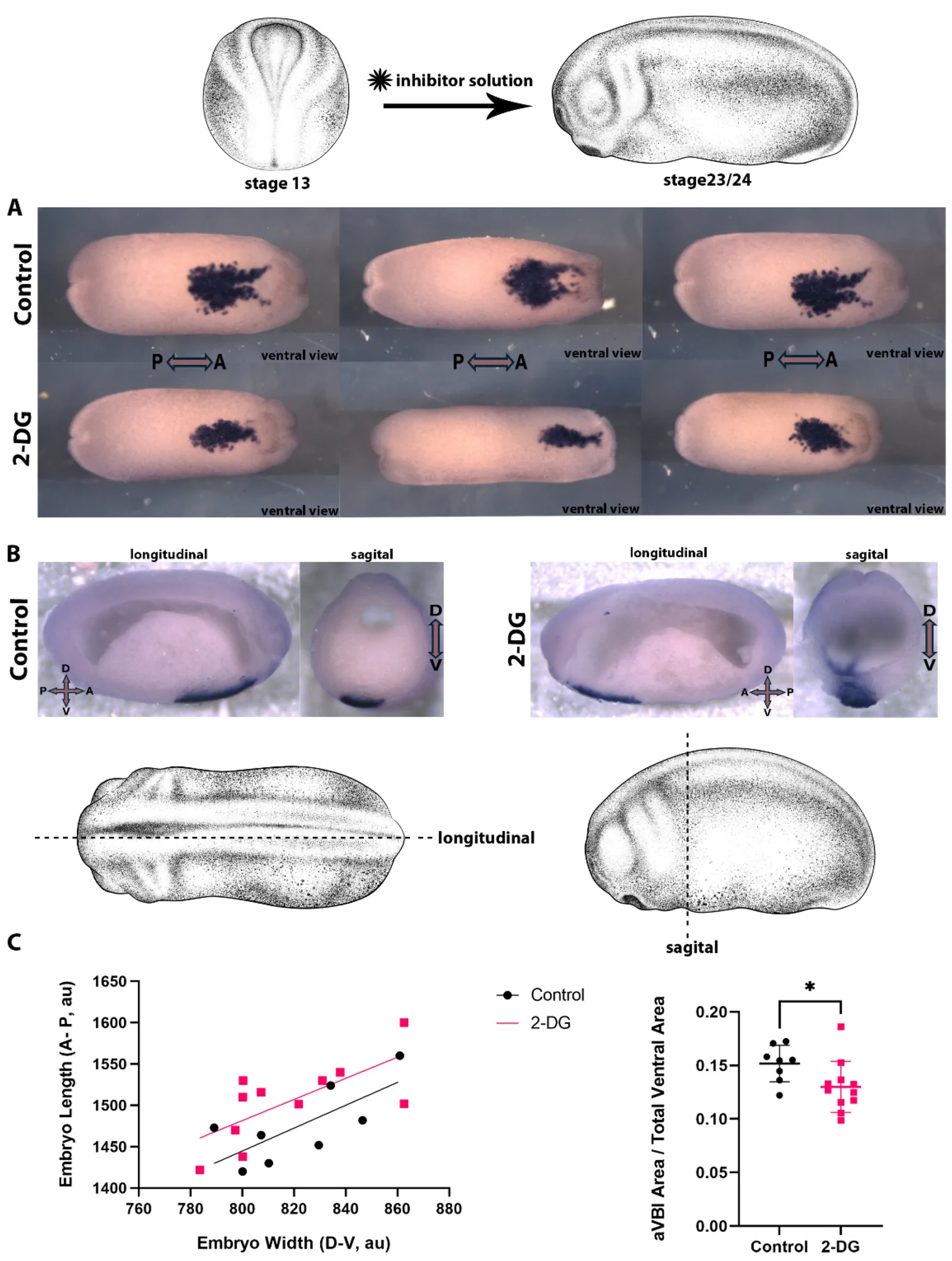
Pharmacological inhibition of glucose-metabolic pathways does not grossly alter the spatial distribution of spib-positive PMC progenitors at the specification stage. (**A**) *Spib* WMISH in control and 2-DG-treated embryos. (**B**) Longitudinal and sagittal sections of the embryos in (**A**), showing that *spib*-positive cells are retained in deeper tissue layers after 2-DG treatment rather than lost. (**C**) Quantification of PMC domain size and overall embryo proportions in control and 2-DG-treated embryos. Left: simple linear regression of embryo length (A–P) against width (D–V) in control (n = 8) and 2-DG-treated (n = 11) embryos; both groups showed a significant positive correlation (control: Pearson r = 0.73, *p* = 0.041; 2-DG: r = 0.70, *p* = 0.017), and the slope (F(1,15 = 0.02, *p* = 0.883) and intercept (F(1,16 = 4.46, *p* = 0.051) did not differ significantly between groups. Right: aVBI area relative to total ventral embryo area (aVBI/TVA ratio) in control (n = 8) and 2-DG-treated (n = 11) embryos, shown as individual values with mean ± SD (unpaired two-tailed *t*-test, t(17) = 2.206, *p* = 0.041, *). Xenopus illustrations © Reprinted from ref. [36].

To test whether glucose metabolism is functionally required for PMC development, we used pharmacological inhibitors that target glucose metabolism upstream of its branch points (2-deoxy-D-glucose, 2-DG) or downstream, within glycolysis (YZ-9), the PPP (6-AN) or the HBP (ST045849) (Figure 1). Embryos treated with each inhibitor from pre-specification (stage 13) through completion of colonization (stage 23/24) developed normally and showed *spib* expression with a spatial pattern comparable to controls, indicating that none of the four inhibitors grossly disrupts PMC fate specification (Figure 5A) (Figure S1 for YZ-9,6-AN and ST045849). For 2-DG specifically, we quantified this further. Histological sectioning of 2-DG-treated embryos confirmed a modest reduction in surface-visible *spib*-positive cells due to incomplete translocation of PMCs to their superficial positions, rather than to a loss of cell number (Figure 5B). However, morphometric quantification of the spib-positive domain revealed that the aVBI area relative to total ventral embryo area (aVBI/TVA ratio) was modestly reduced in 2-DG-treated embryos relative to controls (Figure 5C). This reduction in surface domain area, together with the deeper retention of spib-positive cells observed by sectioning, is consistent with a more compact, less superficially distributed PMC population rather than a failure of specification or loss of cells. The lack of specification phenotype was consistent when embryos were treated with YZ-9, 6-AN or ST045849 (Figure S1). Additionally, to rule out generalized toxicity upon inhibitor treatment we quantified embryo length and width in stage 24 embryos. Embryo length and width remained positively correlated in both control (Pearson r = 0.73, *p* = 0.041, n = 8) and 2-DG-treated embryos (r = 0.70, *p* = 0.017, n = 11) (Figure 5C), and simple linear regression showed no significant difference between groups indicating that overall embryo proportions were unaffected and arguing against a generalized developmental delay as the basis for this phenotype.

**Figure 6:**
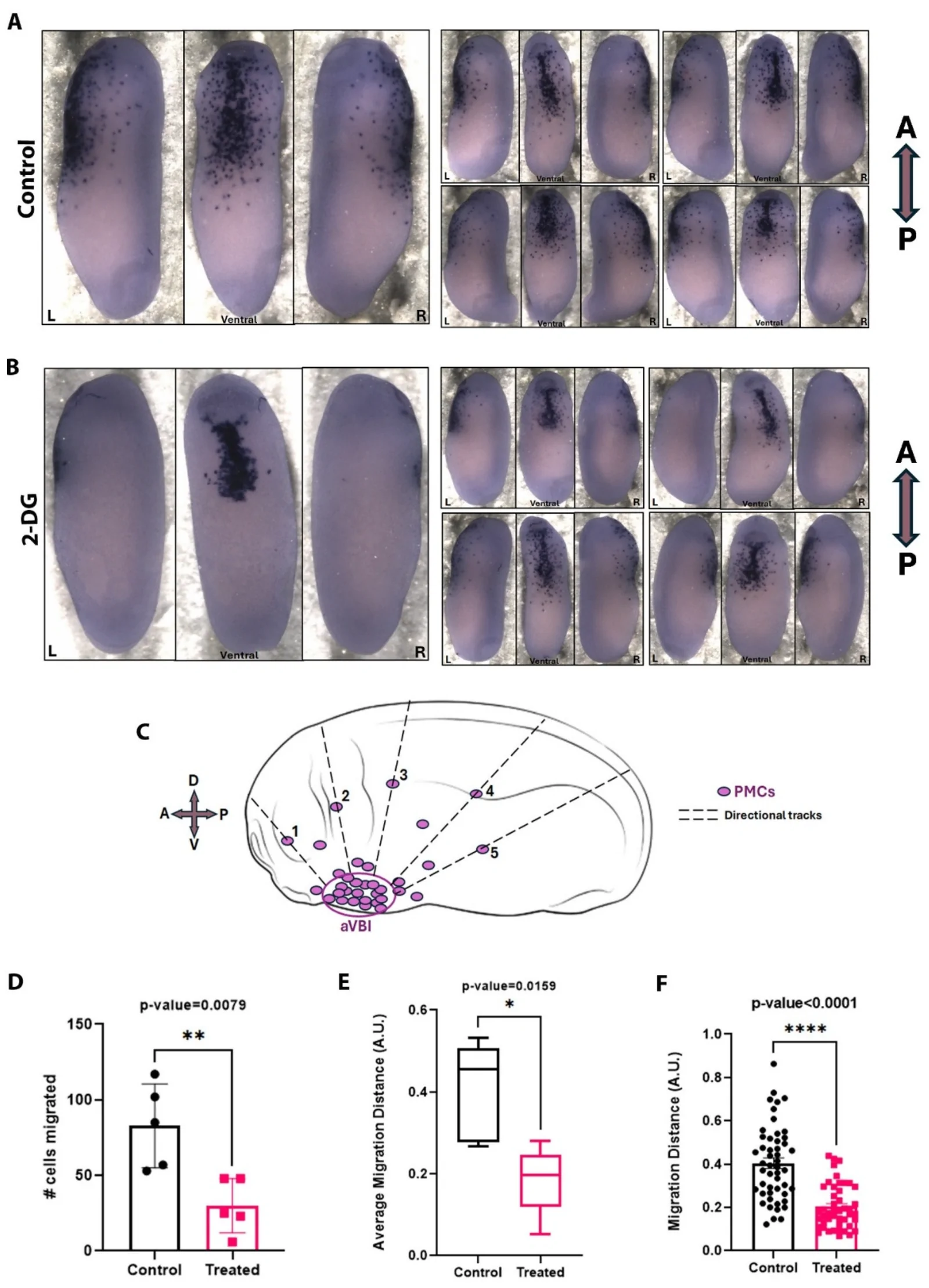
Global inhibition of glucose metabolism impairs PMC colonization. (**A**,**B**) *Spib* WMISH in control (**A**) and 2-DG-treated (**B**) stage 24 embryos. (**C**) Quantification schematic: cells migrating beyond a defined region of interest (ROI) around the anterior ventral blood islands (aVBIs) were counted, and the migration distances of five leading cells per embryo (ROI 1–5) were measured along directional tracks. (**D**) Number of migrated cells per embryo (Mann–Whitney, ** *p* =0.0079). (**E**) Average migration distance per embryo (Mann–Whitney, * *p* = 0.0159). (**F**) Distribution of individual leader-cell migration distances (Mann–Whitney, **** *p* < 0.0001).

In contrast, global inhibition of glucose metabolism markedly impaired PMC colonization of the embryo. Relative to controls, 2-DG-treated embryos showed both fewer PMCs exiting the aVBIs (mean 30 vs. 83 cells/embryo; *p* = 0.0079) and a shorter relative migration distance of the leading cells (mean relative distance 0.20 vs. 0.40; *p* = 0.0159 for per-embryo averages, *p* < 0.0001 for individual leader cells) (Figure 6). Morphological staging confirmed that this phenotype was not attributable to developmental delay. These results show that glucose metabolism is dispensable for PMC specification, with only a modest effect on the size of the aVBIs, but is required for efficient exit and migration of PMCs away from their site of origin.

### 2.3. Glucose Metabolism Controls the Migration of PMCs in Response to Wounding

**Figure 7:**
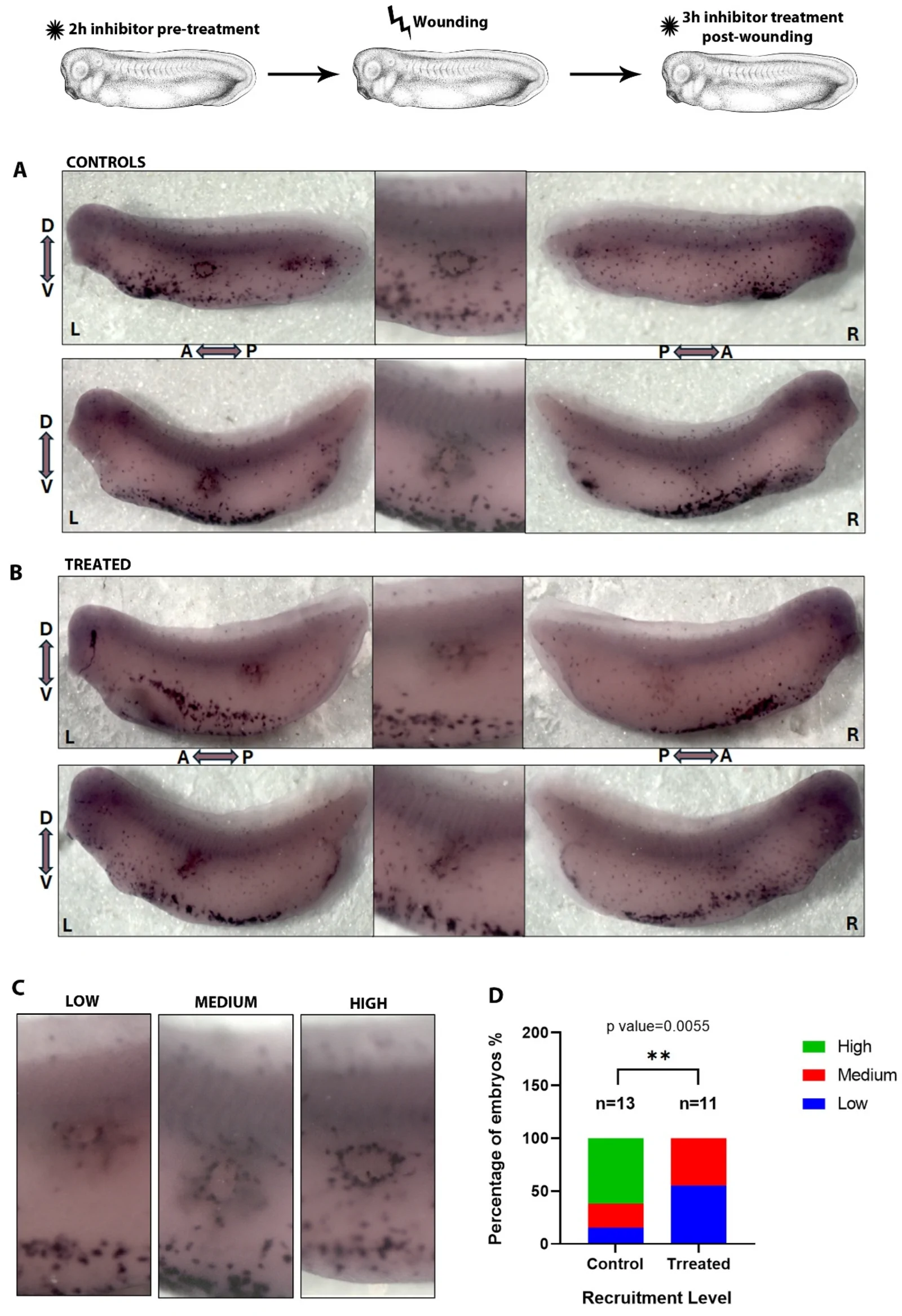
Glucose metabolism is required for efficient PMC recruitment to wounds. (**A**,**B**) *Spib* WMISH of control (**A**) and 2-DG-treated (**B**) stage 32 embryos following wounding. (**C**) Classification of recruitment efficiency into low/medium/high categories. (**D**) Quantification of recruitment levels across treatment groups, expressed as percentages of embryos falling into each category (chi-square test, ** *p* = 0.0055). Xenopus illustrations © Reprinted from ref. [36].

PMCs are recruited rapidly to sites of injury, a hallmark response of their role as the earliest embryonic immune effectors [37]. To determine whether glucose metabolism is similarly required for this response, we wounded embryos in the presence or absence of metabolic inhibitors and scored PMC recruitment to the wound margin by WMISH 3 h later. Global inhibition of glucose metabolism with 2-DG significantly reduced recruitment, shifting the distribution of embryos toward the low/medium recruitment categories relative to controls (*p* = 0.0055, chi-square test) (Figure 7). This data suggests that glucose metabolism is essential for PMC recruitment to wounds.

**Figure 8:**
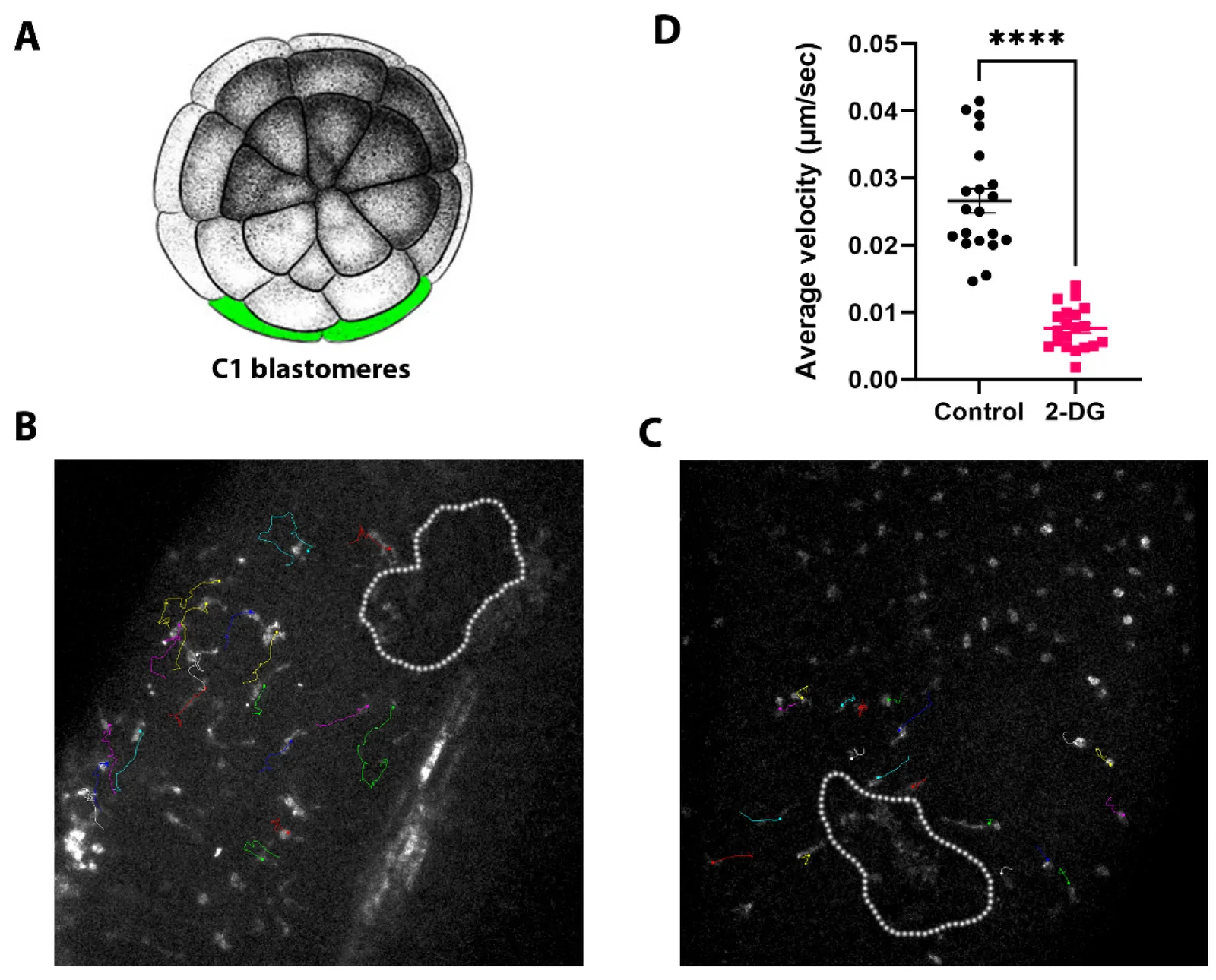
Live imaging reveals reduced PMC migration velocity toward wounds under 2-DG treatment. (**A**) Schematic of memRFP mRNA injection at the C1 blastomere (32-cell stage—animal view) to label the primitive myeloid lineage. (**B**,**C**) Representative still frames from time-lapse movies at the end of imaging, showing memRFP-labelled PMCs with migration tracks approaching the wound margin (dashed outline) in a control (**B**) and a 2-DG-treated (**C**) stage 32 embryo. (**D**) Average migration velocity of PMCs (μm/sec) in control (n = 20) and 2-DG-treated (n = 20) embryos (unpaired *t*-test with Welch’s correction, t(22.66) = 9.75, **** *p* < 0.0001). Xenopus illustrations © Reprinted from ref. [36].

To complement this static, WMISH-based assessment of recruitment with a dynamic readout of PMC behaviour during the wound response, we performed live imaging of PMCs. To achieve this, embryos were injected with membrane-targeted RFP (memRFP) mRNA at the C1 blastomere at the 32-cell stage to specifically label PMCs [38]. Subsequently, embryos were wounded and imaged live by time-lapse microscopy. Using manual cell tracking, we measured the migration velocity of PMCs approaching the wound margin in control and 2-DG-treated embryos (Figure 8). PMCs in 2-DG-treated embryos migrated at a markedly reduced velocity relative to controls, providing direct, dynamic confirmation that glucose metabolism is required for efficient PMC migration during the wound response.

**Figure 9:**
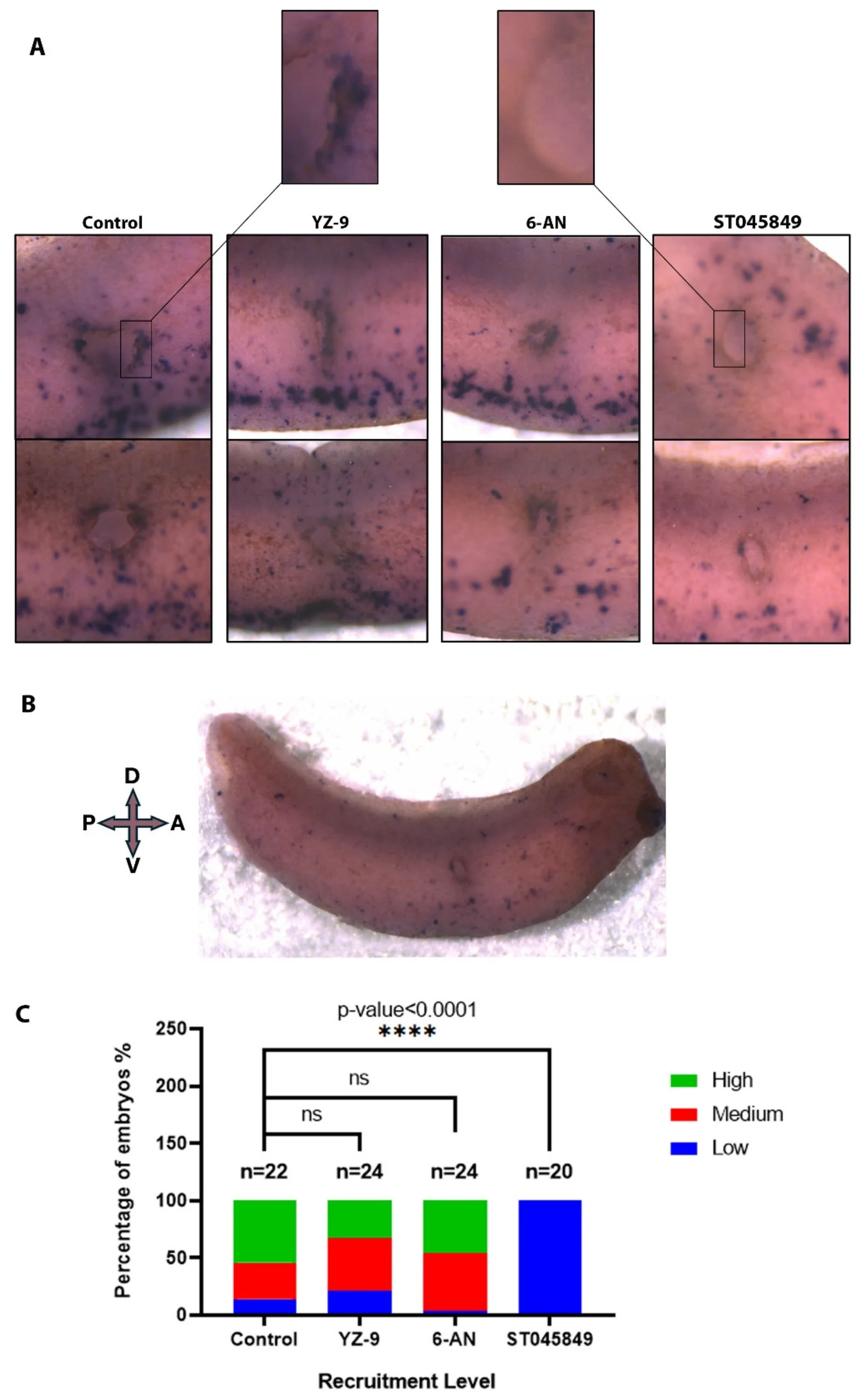
Inhibition of the hexosamine biosynthetic pathway (HBP), but not of downstream glycolysis or the pentose phosphate pathway (PPP), disrupts PMC recruitment to wounds. (**A**) Representative close-ups of wound sites in stage 32 control embryos and embryos treated with YZ-9, 6-AN or ST045849. (**B**) WMISH image showing reduced PMC numbers in an ST045849-treated tadpole. (**C**) Quantification of recruitment efficiency across treatment groups (chi-square test; only ST045849 differed significantly from control, **** *p* < 0.0001).

When individual downstream pathways were tested, inhibition of the glycolysis (YZ-9) or the PPP (6-AN) branch produced only modest, non-significant shifts in recruitment efficiency. In striking contrast, HBP inhibition with ST045849 essentially abolished wound recruitment and additionally reduced the overall number of detectable PMCs (Figure 9). None of the pathway-specific inhibitors grossly altered PMC specification (Figure S1), indicating that these recruitment phenotypes reflect defects in migration or survival rather than in cell fate. Together, these data identify the HBP as a pathway selectively required for PMC recruitment to wounds, distinct from the broader requirement for glucose metabolism in colonization.

## 3. Discussion

Using a combination of single-cell transcriptomic analysis, spatial gene expression, and pharmacological loss-of-function approaches, we show that *Xenopus* PMCs deploy a glucose-metabolic gene programme, spanning glycolysis, the PPP and the HBP, during the developmental window in which they colonize the embryo (Figure 2, Figure 3 and Figure 4). Global inhibition of glucose metabolism impaired both colonization and wound-induced recruitment of PMCs, with only a modest effect on the size of the aVBIs and no effect on PMC number or identity (Figure 5, Figure 6 and Figure 7),while selective inhibition of the HBP, but not of downstream glycolysis or the PPP, reproduced and even exceeded this recruitment defect (Figure 9). These findings indicate that glucose metabolism acts as a regulatory hub for PMC migratory behaviour in vivo, in an embryonic context that precedes the establishment of a functional vasculature.

The dependence of PMC migration on glucose metabolism mirrors emerging paradigms in immunometabolism and developmental cell migration. Embryonic neural crest cells elevate glycolysis prior to delamination to drive epithelial-to-mesenchymal transition via YAP/TEAD signalling [33], and mammalian macrophages rely on glycolysis for efficient recruitment to sites of inflammation, with highly motile M1-like macrophages favouring aerobic glycolysis to fuel rapid ATP generation and biosynthesis during chemotaxis [39,40]. Our observation that global glycolytic blockade (2-DG) impairs PMC migration is consistent with a ‘fuelling the cytoskeleton’ model in which glycolytic flux is spatially coupled to actin-rich protrusive activity at the leading edge [8].

Notably, inhibition of glycolysis downstream of hexokinase (YZ-9) or of the PPP (6-AN) alone produced only modest, non-significant effects on wound recruitment, whereas HBP inhibition (ST045849) essentially abolished it. This is compatible with reports that redox and biosynthetic demands during migration can be met by multiple, partially redundant glycolytic branches. For example, neutrophils divert glycolytic intermediates into a cyclic pentose pathway during the oxidative burst, and PPP blockade alone only partially suppresses superoxide production [41,42], whereas in regenerating *Xenopus* tissue, PPP flux, rather than glycolysis itself, appears to be selectively required for proliferation [43]. The HBP-dependent phenotype we observe more likely reflects a distinct, less redundant requirement. UDP-GlcNAc, the end-product of HBP, is the obligate donor for O-GlcNAcylation, which dynamically regulates the stability and activity of transcription factors, kinases and chemotactic signalling components [44]. Pharmacological O-GlcNAc transferase (OGT) inhibition reduces proliferation and viability in other cell types [45] and can trigger necroptosis in immune cells [46], and genetic loss of OGT is embryonic lethal. Altered O-GlcNAcylation delays wound closure in diabetic skin models [47], together suggesting that HBP flux and O-GlcNAcylation are broadly required across developmental and wound-healing contexts, potentially through effects that extend beyond PMCs themselves.

Similar metabolic dependencies have been reported in other vertebrate models of embryonic myeloid migration. In zebrafish, macrophage recruitment during fin regeneration requires glycolytic and lactate metabolism genes [48]. In particular, zebrafish also possess an early, vasculature-independent population of primitive macrophages that colonize the cephalic mesenchyme, brain, retina and epidermis through an invasive, amoeboid mode of migration before the vasculature is established, resembling the route taken by *Xenopus* PMCs [49]. Macrophage recruitment in later regenerative contexts such as fin regeneration, by contrast, occurs after the vascular network has formed and can rely on vasculature-associated cues [50]—indicating that vasculature-(in)dependence in zebrafish myeloid migration is stage- and context-specific rather than a fixed feature of the lineage. In gastrulating mouse embryos, HBP inhibition disrupts primitive-streak formation whereas blocking late glycolysis does not prevent mesoderm migration [34], echoing our finding that ancillary glucose-metabolic branches, rather than bulk glycolytic ATP production, can be selectively required for developmental cell movements.

More broadly, our findings are consistent with the view that glucose metabolism and its ancillary branches, in addition to their established role in cancer metabolism, may function as a developmental strategy for regulating cell adhesion, motility and tissue remodelling that could be co-opted by neoplastic cells, including those of neural crest and myeloid origin [51,52,53]. As a genetically and experimentally tractable, pre-vascular model of innate immune cell migration, *Xenopus* PMCs could offer a complementary in vivo system for exploring metabolic regulators of immune cell behaviour and 3D cell migration.

Despite the significance of our findings, this study has several limitations. First, whole-embryo pharmacological treatment cannot distinguish a PMC-autonomous requirement for glucose metabolism from indirect effects mediated by other tissues, for example, altered chemokine or growth factor signalling from the wound margin or surrounding mesenchyme. This is an inherent limitation of systemic small-molecule inhibition, which remains a standard and appropriately conservative first-pass approach in a progenitor population for which cell-autonomous genetic tools are not yet established; resolving this question will require PMC-restricted approaches, such as transplantation of metabolically manipulated donor cells into untreated hosts or mosaic/lineage-restricted knockdown, which we identify as a priority for future work. Second, we did not directly confirm target engagement for each inhibitor (e.g., via metabolite or flux measurements), the concentrations used were instead selected from prior in vitro and in vivo validation of each compound [33,34,35] so the absence of a significant phenotype for YZ-9 and 6-AN cannot be taken as definitive evidence against a role for glycolysis or the PPP. Third, we did not investigate OXPHOS or immune effector functions beyond migration and colonization (e.g., phagocytosis, respiratory burst); these lay outside the scope of the present study, which focused on glucose-metabolic branchpoints and their relationship to PMC movement, but represent clear directions for future work. Finally, rescue experiments (e.g., N-acetylglucosamine supplementation to restore HBP flux) cannot be performed since this molecule is not cell-permeable. Future work combining live metabolic imaging (e.g., NAD(P)H autofluorescence or genetically encoded sensors), genetic knockdown of individual metabolic enzymes, extracellular flux analysis (e.g., Seahorse assays) or isotope tracing on isolated PMCs, single-cell metabolomics, and rescue experiments, will help resolve the precise contribution of each pathway, and its downstream effectors, to PMC migration and survival.

## 4. Materials and Methods

### 4.1. Ethics Statement

All procedures involving *Xenopus laevis* adults and embryos were carried out in accordance with national and institutional guidelines for the use of animals in research and were approved by the National Committee for the Protection of Animals Used for Scientific Purposes of the Republic of Cyprus with the licence number (CY/EXP/PR.L 23/05/2022).

### 4.2. Animals, In Vitro Fertilization and Embryo Culture

Ovulation was induced in adult female *Xenopus laevis* by injection of human chorionic gonadotropin (750 U) into the dorsal lymph sac ~16 h before egg collection; eggs were fertilized in vitro using macerated testis fragments, following established husbandry procedures [54]. Testes were harvested by dissection from male *X. laevis* frogs that had been euthanized in 0.06% benzocaine for 30 min and were stored in L-15 medium (10% Fetal Calf Serum, L-Glutamine and 50 mg/mL Gentamycin) at 4 °C until use. Eggs were released from primed females by gentle synchronous lateral and vertical abdominal massage and were fertilized in vitro by mixing them with a homogenized fragment of testis in a glass Petri dish containing 1/3× Marc’s Modified Rogers (MMR) solution. Successful fertilization was confirmed 30 min post-insemination by the characteristic reorientation of the eggs, with the animal hemisphere facing upwards and the vegetal pole facing downwards [55]. After the first cleavage division, the gelatinous jelly coat surrounding the embryos was removed by gently stirring the embryos in 2% L-cysteine (pH 7.8) for approximately 3 min, until tight embryo clustering was observed. Demucilaged embryos were rinsed at least three times in 1/3× MMR to remove residual cysteine and were subsequently transferred to a 4% Ficoll solution prepared in 1/3× MMR for osmotic protection and to facilitate further handling. Embryos were staged according to the normal table of *Xenopus laevis* development [56].

### 4.3. Molecular Cloning and Riboprobe Synthesis

Antisense digoxigenin (DIG)-labelled riboprobes were generated from IMAGE clone plasmids encoding *spib*, GAPDH, ALDOA, GPI and SLC2A3 (Table 1). Plasmid DNA was purified from bacterial cultures using the Plasmid Midi Kit (Qiagen, Hilden, Germany), linearized with Sal I as listed in Table 1, and purified by phenol–chloroform extraction and isopropanol precipitation. Linearized templates were transcribed in vitro with T7 RNA polymerase in the presence of a DIG-labelling mix, treated with TURBO DNase, and probes were purified by LiCl/ethanol precipitation [57,58].

### 4.4. Whole-Mount In Situ Hybridization

WMISH was performed as described [57,59], using SPIB as a PMC-specific marker [31]. Embryos were fixed in 1× MEMFA (2 h, room temperature) and stored in 100% methanol at −20 °C, hybridized with DIG-labelled antisense probes, and probe binding was detected with an alkaline-phosphatase-conjugated anti-DIG antibody and visualized with BM Purple. Embryos were imaged on a Zeiss SteREO Discovery.V12 stereomicroscope (Carl Zeiss AxioVision Rel.8; Carl Zeiss AG, Oberkochen, Germany).

### 4.5. Pharmacological Inhibition and Wounding Assay

Glucose metabolism was inhibited using 2-deoxy-D-glucose (2-DG; 2 mM) [33], the PFKFB3 inhibitor YZ-9 (10 μM), 6-aminonicotinamide (6-AN; 5 μM) [34], and the OGT inhibitor ST045849 (5 μM) [35], each prepared in 0.1× MMR. All concentrations were selected from prior established in vitro or in vivo validation of each compound’s efficacy and specificity [33,34,35] rather than titrated de novo in *Xenopus* PMCs. For specification assays, embryos were treated continuously from stage 13 to stage 23/24 after vitelline membrane removal [60]. For wounding assays, embryos were pre-treated for 2 h, anesthetized in 0.01% benzocaine in the same inhibitor solution, wounded using a Gastromaster (Xenotek Engineering, Bellevue, WA, USA) to generate electrically induced microcurrent lesions, and returned to fresh inhibitor solution for a further 3 h to permit PMC recruitment before fixation for WMISH. Control embryos were treated in parallel with the equivalent volume of vehicle (DMSO) used to dissolve each inhibitor.

### 4.6. Bioinformatics, Image Preparation, Analysis and Statistics

Single-cell RNA-sequencing annotations for Xenopus tropicalis were obtained from the Xenopus Jamboree cell-type atlas (https://xenopus.hms.harvard.edu/) and used without further reprocessing [32]. For colonization assays, the number of *spib*-positive cells exiting a defined region of interest around the aVBIs, and the migration distance of the five most distal (‘leader’) cells per embryo along predefined directional tracks, were quantified in Fiji and expressed as a relative migration index (0–1), after being normalized to the maximum possible migration distance for that track. For wounding assays, recruitment to the wound margin was scored into low/medium/high recruitment efficiency categories, based on the approximate visual density of *spib*-positive cells at the wound site. For specification assays, embryo length (anterior–posterior axis) and width (dorsal–ventral axis) were measured from ventral-view brightfield images in Fiji, and the spib-positive aVBI domain area was measured relative to the total ventral embryo area (aVBI/TVA ratio) by manual outlining of the spib-positive domain and the ventral embryo outline, respectively. Statistical analyses were performed in GraphPad Prism 9.0 using the Mann–Whitney test (colonization), the chi-square test (recruitment categories), Pearson correlation and simple linear regression (embryo length versus width), or an unpaired two-tailed *t*-test after confirming equal variances by an F-test (aVBI/TVA ratio); *p* < 0.05 was considered significant; *p* < 0.05 was considered significant. Illustrations were prepared with BioRender.

All embryo illustrations were obtained from Xenbase (www.xenbase.org RRID:SCR_003280 [36], © Natalya Zahn (2022).

### 4.7. Microinjections and Live Imaging of PMCs

Capped mRNA was in vitro transcribed using mMessage machine kits (Invitrogen, Thermo Fisher Scientific, Waltham, MA, USA ) as described previously [61]. Embryos were injected while being kept in a 4% Ficoll solution in 1/3× MMR. Embryos were injected at the 32-cell stage, according to Nieuwkoop and Faber [56], at the C1 blastomere, which gives rise to the primitive myeloid lineage [38]. Experiments were conducted on both control embryos and embryos treated with 2-DG; the inhibitor treatment was performed as previously described for the wounding assay. Injected embryos were reared in 0.1× MMR. Once embryos reached the appropriate stage, they were wounded as described in Section 4.5 and imaged live by time-lapse microscopy [62], using a Zeiss LSM 710 confocal microscope (Carl Zeiss Microscopy GmbH, Jena, Germany) with a 10× objective lens, capturing one image every 3 min to visualize memRFP-labelled PMCs approaching the wound. PMC migration was tracked manually using the Manual Tracking plugin in Fiji [63], and the average migration velocity was calculated for each embryo from the resulting tracks.

## 5. Conclusions

This study identifies glucose metabolism as a key regulator of PMC behaviour during *Xenopus laevis* embryogenesis, bridging developmental biology and immunometabolism in a system that operates before the establishment of a functional vasculature. Integrating single-cell transcriptomic analysis, spatial gene expression and pharmacological loss-of-function approaches, we show that PMCs express a glucose-metabolic gene programme spanning glycolysis, the PPP and the HBP; that this metabolic activity is dispensable for PMC specification—reducing the size, but not the presence or spatial pattern of the aVBIs—while being required for the colonization of the embryo; and that, among its downstream branches, the HBP is selectively required for PMC recruitment to wounds. Because these results were obtained using whole-embryo pharmacological treatment, they establish a requirement for glucose metabolism in PMC-mediated tissue colonization without yet distinguishing PMC-autonomous from indirect, tissue-level contributions. These findings nonetheless position glucose metabolism and the HBP in particular as an early checkpoint for embryonic innate immune cell function, and establish Xenopus PMCs as a tractable, vasculature-independent model for dissecting the metabolic control of immune cell migration.

## Figures and Tables

**Table 1:** Plasmids and restriction enzymes used for antisense riboprobe synthesis.

Gene	IMAGE Clone	Restriction Enzyme (Antisense)
Spib	5537169	Sal I
SLC2A3	5570438	
GAPDH	5571163	
ALDOA	5537288	
GPI	5511859	

## Data Availability

The authors declare that all data supporting the findings of this study are available within the article and its supplementary information files. All data can be provided by the corresponding authors upon request.

## Supplementary Materials

The following supporting information can be downloaded at: https://www.mdpi.com/article/10.3390/ijms27188175/s1.

## Abbreviations

The following abbreviations are used in this manuscript:

2-DG	2-Deoxy-D-glucose
6-AN	6-Aminonicotinamide
ATP	Adenosine Tri-Phosphate
aVBIs	Anterior Ventral Blood Islands
ECM	Extra-Cellular Matrix
HBP	Hexosamine Biosynthetic Pathway
OGT	O-GlcNAc transferase
OXPHOS	Oxidative Phosphorylation
PMCs	Primitive Myeloid Cells
PPP	Pentose Phosphate Pathway
ROI	Region Of Interest
WMISH	Whole Mount In Situ Hybridization
